# Association of oXiris^®^ Therapy with Lower Vasopressor Requirements and Modulation of Hemodynamic, Inflammatory, and Perfusion Markers in Septic Shock: A Retrospective Cohort Study

**DOI:** 10.3390/jpm15120626

**Published:** 2025-12-14

**Authors:** Nazrin Bakhshaliyeva, Fernando Ramasco Rueda, Ana Estiragués Barreiro, Miguel Ángel Olmos Alonso

**Affiliations:** Department of Anesthesiology and Reanimation, Hospital Universitario de la Princesa, 28006 Madrid, Spain; ana.estiragues@salud.madrid.org (A.E.B.); miguelangel.olmos@salud.madrid.org (M.Á.O.A.)

**Keywords:** septic shock, oXiris^®^, personalized medicine, vasopressors, hemoadsorption

## Abstract

**Background**: Septic shock remains a critical challenge with high mortality, particularly in refractory cases requiring high doses of vasopressors. Hemoadsorption with the oXiris^®^ membrane, capable of simultaneously removing endotoxins, cytokines, and damage-associated molecular patterns (DAMPs), represents a personalized therapeutic strategy targeting the underlying pathophysiology. However, clinical evidence on its impact remains limited and lacks consensus. This study aims to analyze the effects of oXiris^®^ therapy on hemodynamic, inflammatory, and perfusion parameters in a real-world cohort of patients with septic shock. **Methods**: We conducted a retrospective cohort study in a surgical Intensive Care Unit (ICU) at a tertiary hospital, including 45 adult patients with septic shock treated with continuous renal replacement therapy using the oXiris^®^ membrane for at least 48 h. The institutional protocol involved filter changes at least every 24 h during the first 48 h of therapy. Hemodynamic variables, vasopressor doses, and biochemical markers were collected at baseline (T0), 24 h (T1), and 48 h (T2). The primary objective was to describe the evolution of these parameters. Secondary objectives included analysis of 30-day mortality and identification of prognostic factors. **Results**: The cohort consisted of 45 patients (80.0% male, median age 71 years), with a predominance of abdominal infectious focus (71.1%). A significant reduction in median norepinephrine requirements was observed from T0 to T2 (*p* < 0.00001), along with a significant increase in mean arterial pressure (MAP) (*p* < 0.00001). Key markers of perfusion and inflammation also improved, with a significant decrease in arterial lactate (*p* < 0.00001) and procalcitonin (*p* = 0.00082) at 48 h. No significant changes were observed in the Sequential Organ Failure Assessment (SOFA) score. The observed mortality rate in the ICU was 31.1%, lower than the median predicted mortality by Simplified Acute Physiology Score II (SAPS II) (37%). Baseline Charlson Comorbidity Index (CCI), creatinine, arterial lactate, and SOFA score were independent predictors of mortality. **Conclusions**: In this cohort of septic shock patients, therapy with oXiris^®^, applied with a frequent filter exchange protocol, was associated with a significant reduction in vasopressor requirements and an improvement in key hemodynamic, perfusion, and inflammatory markers. The observed ICU mortality was lower than predicted by severity scores. These findings support the role of oXiris^®^ as a personalized adjuvant therapy in specific septic shock phenotypes and underscore the need for prospective randomized trials to confirm these benefits.

## 1. Introduction

Sepsis represents a condition of high morbidity and mortality, affecting approximately 48.9 million people globally each year and positioning itself as one of the leading causes of in-hospital death [1]. Its mortality ranges from 10 to 15% in cases of sepsis to as high as 40–50% in septic shock [2].

To establish diagnostic criteria, the Third International Consensus Definitions for Sepsis and Septic Shock (Sepsis-3) define sepsis as the presence of organ dysfunction, identified by an increase of ≥2 points in Sequential Organ Failure Assessment (SOFA) score [3]. Septic shock is characterized by arterial hypotension requiring vasopressor support to maintain a mean arterial pressure ≥ 65 mmHg, accompanied by hyperlactatemia > 2 mmol/L, despite adequate fluid resuscitation [4].

One of the fundamental pathophysiological mechanisms in sepsis is the disproportionate activation of proinflammatory cascades triggered by pathogen-associated molecular patterns (PAMPs), such as lipopolysaccharides from Gram-negative bacteria, and damage-associated molecular patterns (DAMPs) [5]. This dysregulated systemic inflammatory response, characterized by the overproduction of cytokines like TNF-α and interleukins 1, 6, and 10, contributes to the development of multiple organ dysfunction [4].

In this pathophysiological context, the theory by Anand Kumar [5] underscores the importance of bacterial load and endotoxin as the main drivers of organ dysfunction in septic shock [6]. According to this model, early reduction in this bacterial burden—through optimized antibiotic therapy and strategies for endotoxin and cytokine adsorption—is crucial for improving outcomes, as once established, shock can become irreversible or perpetuated if not promptly reversed [7].

Current treatment for septic shock is primarily based on hemodynamic resuscitation, infectious source control, and antibiotic administration [8]. The importance of optimal antimicrobial therapy through an “antibiotic stewardship” approach is a fundamental pillar for reducing bacterial load as early as possible and preventing disease progression [5]. However, the high heterogeneity of sepsis—with variations in age, comorbidities, infecting microorganism, and genetic predisposition—suggests that a “one-size-fits-all” approach is unlikely to succeed [6,9]. Consequently, there is a growing trend towards the personalization of resuscitation, seeking specific hemodynamic phenotypes to guide interventions [10]. An example of this approach is the ANDROMEDA-SHOCK-2 trial, which aims to compare a personalized resuscitation strategy based on clinical phenotyping and peripheral perfusion assessment (such as capillary refill time, CRT) against the standard of care in early septic shock [11,12].

Despite these advances in hemodynamic resuscitation and antibiotic management, mortality from septic shock remains alarmingly high, especially in refractory cases [13]. Refractory septic shock has been defined with norepinephrine thresholds ranging from 0.2 µg/kg/min to 3.8 µg/kg/min in various studies [7,14]. Norepinephrine doses around 1 µg/kg/min have been associated with mortality rates exceeding 80%, and one retrospective study found that a dose above 0.5 µg/kg/min corresponded to a mortality of approximately 45% [8,15]. Given this high mortality in situations of refractory shock or high vasopressor needs, there is a crucial interest in exploring other adjuvant therapies, such as blood purification techniques [16].

In this context, hemoadsorption has emerged as an adjuvant therapeutic strategy that specifically targets proinflammatory mediators [17,18]. Among these, the oXiris^®^ filter (Baxter International Inc., Deerfield, IL, USA) is notable for its ability to simultaneously adsorb endotoxins, inflammatory cytokines, and DAMPs, while also maintaining renal support functionality [9,10,19].

There is a growing interest in the use of the oXiris^®^ membrane and other blood purification therapies for treating septic shock, especially in refractory situations [12,20]. Nevertheless, despite emerging evidence, a notable lack of scientific consensus persists regarding its clinical efficacy and the optimal timing of its application [8,13,19].

Therefore, this study aims to analyze the effects of hemoadsorption with the oXiris^®^ membrane on hemodynamics, perfusion and inflammatory biomarkers, and mortality in patients with septic shock. Through a retrospective analysis, we studied the hemodynamic and biochemical parameters of patients who received hemoadsorption with oXiris^®^ during the first 48 h from the start of therapy. As a secondary objective, we evaluate 30-day mortality.

## 2. Materials and Methods

We conducted a retrospective study at a tertiary hospital in Madrid, focusing on patients diagnosed with perioperative septic shock admitted to the surgical Intensive Care Unit (ICU) between January 2019 and December 2024. These patients were treated with continuous renal replacement therapy (CRRT) using the oXiris^®^ membrane.

In all cases, the oXiris^®^ membrane was used in the context of veno-venous continuous hemodiafiltration (CVVHDF), which represents the standard CRRT modality in our institution. This approach combines convective and diffusive clearance, enabling effective solute removal while simultaneously enhancing the hemoadsorptive function of the oXiris^®^ membrane. The membrane’s unique design incorporates a polyethyleneimine (PEI) coating that provides a strong positive surface charge, allowing for the adsorption of negatively charged endotoxins, as well as a range of inflammatory mediators including cytokines, DAMPs, and PAMPs. According to our unit’s protocol for the management of septic shock, the oXiris^®^ membrane was replaced every 24 h during the first 48 h of therapy to preserve its adsorptive capacity.

Data were collected from hospital records and computerized monitoring sheets recorded in the medical software (HCIS). The study was approved by the Medical Research Ethics Committee under the assigned number 5390 on 11 January 2024.

We included adult patients (≥18 years) with a diagnosis of septic shock according to established international criteria, who received CRRT with the oXiris^®^ hemofilter for a minimum of 48 h. The 48 h threshold was selected to ensure sufficient exposure to the therapy and to enable evaluation of hemodynamic, biochemical, and inflammatory responses. Patients who died within the first 24 h or did not complete the initial treatment cycle were excluded to avoid confounding results due to very early mortality unrelated to treatment effect. This approach aimed to provide a more accurate assessment of the potential impact of oXiris^®^ therapy over a complete and evaluable course.

The primary objective was to describe the evolution of hemodynamic parameters, vasoactive drug doses, and markers of perfusion and systemic inflammation during the first 48 h of hemoadsorption with the oXiris^®^ filter in patients with septic shock. Secondary objectives included evaluating outcomes by subgroups, such as the source of infection (abdominal vs. non-abdominal) and Simplified Acute Physiology Score II (SAPS II score), and identifying factors related to mortality.

Variables were analyzed at three time points: T0 (baseline), T1 (24 h after therapy initiation), and T2 (48 h after therapy initiation). The analyzed variables included hemodynamic parameters such as systolic arterial pressure (SBP), mean arterial pressure (MAP), diastolic blood pressure (DBP), and the PaO_2_/FiO_2_ ratio (P/F ratio); biochemical markers such as creatinine, lactate, procalcitonin, and urea; severity scores like the SOFA score; and the doses of vasopressors, specifically norepinephrine, epinephrine, and vasopressin.

For repeated measures analysis of renal and metabolic parameters across three time points (T0, T1, and T2), we used the Friedman test. When a statistically significant global difference was detected (*p* < 0.05), post hoc pairwise comparisons were performed using mean rank differences. A comparison was considered statistically significant if the observed difference exceeded the predefined minimum threshold (Minimum required difference in mean rank). To avoid redundancy, individual *p*-values for each pairwise comparison were not reported in the text but are detailed in the results table and statistical output.

Therapeutic success criteria were established based on a norepinephrine reduction of at least 30% at 24 and 48 h, and a lactate reduction of at least 20% at 24 h and 40% at 48 h. Both criteria were chosen to provide objective and quantifiable thresholds for a structured evaluation of the therapy’s effectiveness within a retrospective framework. These criteria reflect key indicators of hemodynamic and metabolic response widely recognized in septic shock resuscitation and are consistently evaluated in the scientific literature to monitor the reversal of tissue hypoperfusion and the reduction in vasopressor dependence.

Continuous variables were expressed as mean ± standard deviation (SD) or median with interquartile range (IQR), and discrete variables as frequencies and percentages. The Shapiro–Wilk test was used to assess normality, the Mann–Whitney U test for continuous variables, the Chi-square test for discrete variables, and the Friedman test for temporal comparisons. Survival analysis was performed using the Log-rank test, and ROC curves were used for predictive variables. A *p*-value of *p* < 0.05 was considered statistically significant.

Statistical analysis was performed using MedCalc^®^ Statistical Software v23.2.1.

## 3. Results

### 3.1. Baseline Characteristics

From an initial sample of 643 patients, 45 met the inclusion criteria and had no exclusion criteria. Initial demographic data showed a predominance of male patients (n = 36, 80.0%), with a median age of 71 years (IQR 63.5–79). Regarding the source of infection, the majority of patients had an abdominal source (n = 32, 71.1%) compared to other sources (n = 13, 28.9%). Among the patients categorized as having ¨other¨ infection source (n = 13), the specific diagnoses included respiratory tract infections (such as bronchoaspiration or post-thoracotomy pneumonia), urinary tract infections (including septic shock from lithiasic uropathy), head/neck region sources (such as odontogenic abscess or mandibular osteonecrosis), and vascular complications (such as infection of an iliac bypass surgical wound). The baseline median values for SAPS II and SOFA scores were 46.00 (IQR 36.50–57.00) and 9.00 (IQR 7.00–10.00), respectively. The detailed characteristics of the sample are described in Table 1.

In the analysis of baseline variables, no significant differences were observed in any of the initial parameters between the abdominal and non-abdominal source groups, including age, Charlson Comorbidity Index (CCI), arterial pressures (SBP, DBP, MAP at T0), PaO_2_/FiO_2_ ratio (P/F ratio at T0), creatinine, arterial lactate, procalcitonin, proBNP, urea, SAPS II, SOFA score and norepinephrine doses.

### 3.2. Temporal Evolution of Key Variables

The following variables were measured at three time points: T0 (baseline), T1 (24 h post-initiation), and T2 (48 h post-initiation). Regarding hemodynamic parameters, a significant increase in SBP was observed at T1 and T2 compared to T0 (*p* = 0.00019), with no significant difference between T1 and T2. Similarly, a significant increase in DBP was found at T1 and T2 compared to T0 (*p* = 0.00467), with no difference between T1 and T2 (*p* = 0.00467). Mean arterial pressure (MAP) also showed a significant increase at T1 and T2 compared to T0 (*p* < 0.00001), with no difference between T1 and T2.

In contrast to the hemodynamic parameters, the PaO_2_/FiO_2_ ratio (P/F ratio) showed no significant differences at any of the analyzed time points (T0, T1, T2).

Regarding renal and metabolic markers, a significant reduction in creatinine was observed at T1 and T2 compared to T0, with a significant difference also found between T1 and T2. Lactate showed a significant reduction at T2 compared to T0 and T1, although no significant difference was identified between T0 and T1. Urea presented a significant reduction at T2 compared to T0 and T1, with a significant difference also between T0 and T1.

Procalcitonin, analyzed in 33 of the 45 cases due to missing values, showed a significant reduction at T2 compared to T0 and T1 (overall *p*-value = 0.00082), with no significant difference between T0 and T1. It is relevant to note that a non-statistically significant increase was observed at T1 (median 6.9 vs. 4.1 at T0).

Concerning severity scores, no significant differences were found in the SOFA score at any of the analyzed time points (T0, T1, T2).

**Figure 1:**
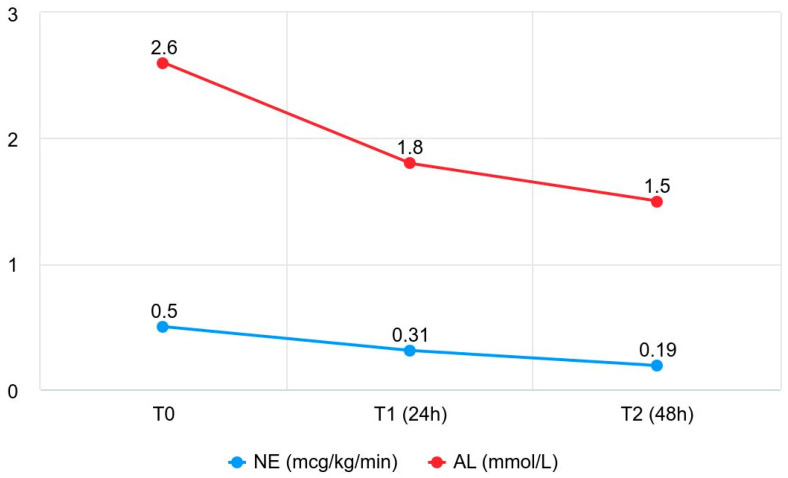
Evolution of median norepinephrine dose and arterial lactate during the first 48 h after initiation of oXiris therapy. Abbreviations: NE, Norepinephrine, AL, arterial lactate; T0, baseline; T1, 24 h after therapy initiation; T2, 48 h after therapy initiation.

A significant reduction in the norepinephrine dose was observed at T2 compared to T0 and T1 (*p* < 0.00001), with a significant difference also recorded between T0 and T1 (*p* < 0.025) (Figure 1). These results are shown in detail in Table 2.

### 3.3. Therapeutic Success Rates

Overall, the therapeutic success rate based on norepinephrine reduction (at least 30%) was 57.8% at T1 (24 h) and 62.2% at T2 (48 h). No significant differences in success rates were found between the abdominal and non-abdominal source groups at either T1 or T2.

For the total population, the success rate based on lactate reduction (≥20% at T1, ≥ 40% at T2) was 44.4% at T1 and 40.0% at T2. No significant differences were observed between the abdominal and non-abdominal source groups for the lactate success criteria at T1 or T2. The success rates are detailed in Table 3.

### 3.4. Mortality Assessment

The overall mortality rate was 48.9% (n = 22). Specifically, 30-day mortality was 26.7% (n = 12) and ICU mortality was 31.1% (n = 14). The median SAPS II-predicted mortality for the total population was 37% (95% CI 0.33–0.48). See Table 4.

### 3.5. Analysis of Subgroups

When comparing by source of infection, the ICU mortality rate was significantly higher in the group with an abdominal source (40.6%) compared to the non-abdominal source group (7.7%) (*p* = 0.0325). See Table 5.

When analyzed by baseline SAPS II (low ≤ 46 vs. high > 46), the ICU mortality rate was significantly higher in patients with SAPS II > 46 (45.5% vs. 17.4%, *p* = 0.0444).

### 3.6. Factors Associated with Mortality

In the analysis of factors associated with mortality, several variables were identified as having a significant association. Specifically, each one-point increase in the Charlson Comorbidity Index (CCI) increased the odds of overall mortality by 52% (Odds Ratio [OR]: 1.5223, 95% CI: 1.1400 to 2.0328; *p* = 0.0044). For 30-day mortality, each one-point increase in the CCI raised the odds by 63% (OR 1.6331, *p* = 0.0079). Baseline creatinine levels (T0) were also associated with higher risk, as each 1 mg/dL increase in baseline creatinine increased the odds of 30-day mortality by 103% (OR 2.0339, *p* = 0.0233).

Baseline arterial lactate levels (T0) were a predictor of ICU mortality: each 1-point increase in baseline lactate increased the odds of ICU mortality by 30.8% (OR 1.3080, *p* = 0.0221). The baseline SOFA score (T0) also correlated with ICU mortality, with each point on the SOFA scale increasing the odds of ICU mortality by 34.5% (OR 1.3446, *p* = 0.0376). Finally, a baseline SAPS II score greater than 46 increased the odds of death in the ICU by 3.96 times (OR: 3.9583, 95% CI: 1.0095 to 15.5205; *p* = 0.0484).

## 4. Discussion

Septic shock remains a critical condition with high mortality, where the personalization of resuscitation and the use of adjuvant therapies are active areas of research. In this context, hemoadsorption with specialized membranes such as oXiris^®^ has emerged as a promising therapeutic strategy to modulate the dysregulated inflammatory response characteristic of this pathology. The unique ability of oXiris^®^ to simultaneously adsorb cytokines and endotoxins allows for the modulation of the dysregulated systemic inflammatory response that leads to vasoplegia [10,14,15]. Specifically, the capacity of oXiris^®^ to adsorb endotoxins is mediated by its polyethyleneimine (PEI) layer, which has positively charged amino groups that bind to the negatively charged endotoxin, achieving up to 68% removal in two hours under experimental in vitro conditions [21]. This mechanism of action has been the basis for its clinical application in patients with sepsis and septic shock [18,19].

Our findings demonstrate a significant improvement in the hemodynamic parameters of patients treated with oXiris^®^. A notable increase in mean, systolic, and diastolic arterial pressure was observed, along with a significant reduction in the dose of vasopressors, specifically norepinephrine and vasopressin, at 48 h from the start of treatment. These results are consistent with the previous literature suggesting that hemoadsorption with oXiris^®^ can improve hemodynamics and reduce the need for vasopressors in patients with septic shock [16,20,22]. A 30% reduction in norepinephrine requirements at 24 and 48 h reflects a significant improvement in hemodynamic stability and a reversal of vasoplegia, which is a primary therapeutic goal in the “decatecholaminization” of septic shock.

Concurrently, a significant reduction in arterial lactate levels was observed at 48 h. This greater than 20% improvement in lactate clearance suggests a recovery from tissue hypoperfusion and an enhancement of cellular oxygenation, which are key pathogenic mechanisms in septic shock. The ability of oXiris^®^ to improve these perfusion parameters has been documented in several previous studies [20,22], reinforcing the importance of considering these outcomes beyond crude mortality.

Regarding markers of the inflammatory response, procalcitonin showed a significant reduction at 48 h in our study. Procalcitonin is considered a biomarker of bacterial infection severity and is typically elevated in Gram-negative sepsis. While this may reflect an underlying endotoxemic component, its role as a direct surrogate marker of endotoxin levels is not established. Rather, high procalcitonin may help identify a phenotype commonly associated with endotoxin-driven inflammation [23,24]. Previous studies have consistently reported the ability of oXiris^®^ to decrease levels of inflammatory biomarkers such as CRP, IL-6, and lactate, along with improvements in organ function [9,25]. We observed higher mortality in patients with an abdominal source of infection compared to other sources. Patients with abdominal sepsis in our series had a lower median procalcitonin value than patients with other sources, although this was not statistically significant, suggesting a lower endotoxin burden in the abdominal sepsis group. The endotoxin burden and its early removal may be important for the prognosis of these patients [26].

Very recent observational studies confirm results similar to ours, showing improvements in hemodynamic, perfusion, and inflammatory parameters, as well as a decreased need for vasopressors [22,25,27]. One of them, a retrospective European study in Poland very similar to ours, investigated the impact of the oXiris^®^ membrane in 32 patients with septic shock. It revealed a statistically significant reduction in the dose of vasopressin after 24 and 72 h, and in norepinephrine after 72 h, suggesting a positive impact on hemodynamic stability. However, no significant changes in procalcitonin or lactate levels were observed, nor was there a beneficial effect on mortality, with 72% of patients dying, which was consistent with the mortality risk predicted by the SAPS II score at admission [22]. Our results are better in a cohort of similar severity, perhaps due to our protocol of changing the oXiris^®^ membrane at least every 24 h during the first 48 h, unlike the Polish study where the membrane was not changed during the first 72 h.

It is important to acknowledge the heterogeneity in the results reported in the literature related to oXiris^®^, which exemplifies the need for studies like ours to accumulate evidence. Two recent systematic reviews, while indicating improvement in hemodynamic and perfusion parameters, underscore the variability in outcomes and the need for more real-world data like our study, as well as high-quality research to better understand the factors that determine treatment response [28,29]. One of the published meta-analyses, which evaluated 14 studies with 695 patients, suggested a possible association between the use of the oXiris^®^ filter and lower 28-day mortality (OR 0.53), but emphasized that the certainty of the evidence was low and that further studies are needed to confirm this finding. This meta-analysis also documented improvements in SOFA score, norepinephrine dose, and IL-6 and lactate levels [28]. In contrast, the other published meta-analysis showed that although PCT, IL-6, and CRP levels in the oXiris^®^ group were significantly lower than in the control group, there were no significant differences in norepinephrine dose, organ function status, or 28-day mortality [29].

The ICU mortality rate in our study was 31.1%, which is lower than the median predicted mortality probability according to the SAPS II score in our population (37%). The overall 30-day mortality was 48.9%. It is worth noting that while SAPS II was originally designed to estimate hospital mortality, in this study it was used as a general severity index at ICU admission. Its association with ICU mortality, as demonstrated in our cohort, is consistent with recent evidence suggesting that SAPS II may retain prognostic value within the ICU setting. This finding suggests that the intervention with oXiris^®^, in the context of our center’s clinical practice, may have been associated with a lower actual mortality than that predicted by the patients’ initial severity. Patients with a higher baseline SAPS II had a greater probability of dying (*p* = 0.0444) [30]. Additionally, baseline Charlson Comorbidity Index (OR: 1.52), creatinine (OR: 2.03), lactate (OR: 1.30), and SOFA score (OR: 1.34) were significant independent predictors of mortality in our study. These prognostic data may be useful in selecting which patients could benefit from rescue therapy with oXiris^®^.

It is important to consider how the success of these interventions is evaluated in the current context of critical care medicine. Authors such as Vincent and others have proposed a revision of the outcomes traditionally used in sepsis and septic shock, arguing for the need to balance guidelines with personalized treatment [31]. They contend that 30-day mortality, while an important endpoint, can be influenced by multiple factors such as the primary disease, comorbidities, and end-of-life decisions, making it a less “clear-cut” endpoint. Specifically regarding the evaluation of adsorption therapies, considering outcomes other than mortality has also been proposed [32].

This study aims to provide relevant real-world data from a European context. However, it has limitations that should be acknowledged. Its non-randomized and retrospective nature, with reliance on historical clinical records, exposes the analysis to confounding variables and potential information bias, as therapeutic decisions were made without a standardized prospective protocol. The sample size, with only 45 patients treated with oXiris^®^, restricts statistical power, especially when the cohort is disaggregated into subgroups by septic focus or organ dysfunction score, leaving each category with too few cases for robust inferences. Furthermore, the absence of a contemporary control group requires comparing observed outcomes with mortality predictions calculated by scores like SAPS II and limits the ability to attribute hemodynamic or biomarker changes exclusively to the action of the oXiris^®^ membrane.

Different approaches to the use of oXiris^®^ have been proposed in the context of septic shock and other scenarios, which can help in decision-making in an environment of high mortality and uncertainty, within the framework of treatment personalization [33,34]. In the future, better methodological consensus based on real-world studies and clinical trials will improve our individualization of treatment with oXiris^®^ [35,36].

This retrospective study evaluates the real-world experience with the oXiris^®^ adsorption membrane in patients with septic shock admitted to a surgical ICU, providing valuable information on its impact on improving hemodynamic parameters, vasopressor needs, and inflammatory and peripheral perfusion biomarkers.

## 5. Conclusions

In conclusion, this retrospective study provides evidence of the impact of hemoadsorption with oXiris^®^ on the improvement of hemodynamic parameters, the reduction in vasopressor doses, and the decrease in inflammatory and perfusion biomarkers, such as procalcitonin and lactate, in patients with septic shock. The observed mortality in the ICU was lower than that predicted by the SAPS II score. Given the retrospective design and the lack of a control group, our findings should be interpreted with caution. The observed hemodynamic and biochemical improvements during the first 48 h of Oxiris^®^ therapy are hypothesis-generating and underscore the need for prospective controlled trials to confirm these preliminary observations.

## Figures and Tables

**Table 1:** Comparison of Baseline Characteristics between Abdominal and Non-Abdominal Sources of Infection.

Variable	Abdominal Source (Median [IQR])	Non-Abdominal Source (Median [IQR])	*p*-Value
Age (years)	71.00 [63.50–79.00] (n = 32)	71.00 [68.00–76.25] (n = 13)	0.9900
CCI	6.00 [2.50–7.00] (n = 32)	5.00 [3.75–6.25] (n = 13)	0.6316
SBP at T0 (mmHg)	100.00 [90.00–110.00] (n = 32)	100.00 [95.00–120.00] (n = 13)	0.5362
DBP at T0 (mmHg)	50.00 [43.50–54.00] (n = 32)	45.00 [40.00–46.25] (n = 13)	0.1113
MAP at T0 (mmHg)	65.84 [59.67–70.00] (n = 32)	61.67 [60.00–73.33] (n = 13)	0.8211
P/F Ratio at T0	244.00 [188.00–299.00] (n = 32)	232.00 [196.75–280.50] (n = 13)	0.9401
Creatinine at T0 (mg/dL)	1.79 [1.06–2.47] (n = 32)	1.87 [1.52–2.73] (n = 13)	0.4156
Arterial Lactate at T0 (mmol/L)	2.85 [1.85–5.30] (n = 32)	2.30 [1.00–3.42] (n = 13)	0.1172
Procalcitonin at T0 (ng/mL)	6.57 [2.02–14.47] (n = 29)	14.00 [0.81–71.02] (n = 13)	0.9025
proBNP at T0 (pg/mL)	1654.00 [788.50–3862.75] (n = 3)	5032.00 [2052.25–23008.00] (n = 3)	0.4000
Urea at T0 (mg/dL)	61.50 [54.00–105.00] (n = 30)	87.00 [45.75–109.75] (n = 13)	0.7609
SAPS II	46.00 [34.50–57.00] (n = 32)	47.00 [37.00–56.25] (n = 13)	0.7828
Predicted Mortality (%)	36.93 [15.97–61.93] (n = 32)	39.19 [19.64–60.30] (n = 13)	0.7828
SOFA at T0	9.00 [7.00–10.50] (n = 32)	8.00 [5.75–9.25] (n = 13)	0.2353
Norepinephrine at T0 (mcg/kg/min)	0.52 [0.30–0.95] (n = 32)	0.42 [0.12–0.85] (n = 13)	0.3667

Abbreviations: CCI, Charlson Comorbidity Index; T0, baseline; SBP, Systolic Blood Pressure; DBP, Diastolic Blood Pressure; MAP, Mean Arterial Pressure; P/F Ratio, PaO_2_/FiO_2_ Ratio; SAPS II, Simplified Acute Physiology Score II; SOFA, Sequential Organ Failure Assessment; IQR, Interquartile Range.

**Table 2:** Evolution of Hemodynamic, Metabolic Parameters, and Vasopressor Doses.

Variable	T0 Median [IQR]	T1 Median [IQR]	T2 Median [IQR]	*p*-Value
SBP (mmHg)	100.00 [90.00–110.00] (n = 45)	110.00 [99.50–119.25] (n = 45)	115.00 [103.75–120.00] (n = 45)	0.00019
DBP (mmHg)	45.00 [40.00–50.75] (n = 45)	50.00 [45.00–59.25] (n = 45)	55.00 [48.00–60.00] (n = 45)	0.00467
MAP (mmHg)	63.33 [60.00–70.83] (n = 45)	70.00 [63.25–75.67] (n = 45)	73.00 [68.67–80.00] (n = 45)	<0.00001
P/F Ratio	232.00 [189.25–298.50] (n = 45)	290.00 [233.50–311.50] (n = 45)	300.00 [201.50–333.25] (n = 45)	0.15031
Creatinine (mg/dL)	1.87 [1.25–2.55] (n = 45)	1.48 [1.04–2.05] (n = 45)	1.22 [0.83–1.53] (n = 45)	<0.00001
Arterial Lactate (mmol/L)	2.60 [1.48–5.05] (n = 45)	1.80 [1.40–3.73] (n = 45)	1.50 [1.20–2.50] (n = 45)	<0.00001
Procalcitonin (ng/mL)	4.10 [1.36–25.81] (n = 33)	6.85 [1.01–31.44] (n = 33)	4.50 * [0.84–21.50] (n = 33)	0.00082
Urea (mg/dL)	71.00 [48.75–105.00] (n = 43)	49.00 [40.00–76.00] (n = 43)	45.00 [27.00–53.75] (n = 43)	<0.00001
SOFA Score	9.00 [7.00–10.00] (n = 45)	8.00 [6.00–10.00] (n = 45)	8.00 [6.00–10.25] (n = 45)	0.19790
Norepinephrine (mcg/kg/min)	0.50 [0.29–0.93] (n = 45)	0.31 [0.08–0.60] (n = 45)	0.19 [0.05–0.37] (n = 45)	<0.00001

Abbreviations: T0, baseline; T1, 24 h after therapy initiation; T2, 48 h after therapy initiation; IQR, Interquartile Range; SBP, Systolic Blood Pressure; DBP, Diastolic Blood Pressure; MAP, Mean Arterial Pressure; P/F Ratio, PaO_2_/FiO_2_ Ratio; SOFA, Sequential Organ Failure Assessment. The *p*-value represents the overall significance of the change across the three time points (Friedman test). * The significant reduction in procalcitonin (PCT) was determined by the Friedman test, which is based on mean ranks (T0: 2.24 vs. T2: 1.48) rather than median values. This non-parametric method was chosen to account for the high inter-individual variability and extreme values present in the dataset.

**Table 3:** Therapeutic Success Rates.

Parameter	Time Point	Criterion Definition	Overall Success (N, %)	Success Abdominal Source (N, %)	Success Non-Abdominal Source (N, %)	*p*-Value
Norepinephrine Dose	T1	≥30% reduction at 24 h	26 (57.8%)	18 (56.2%)	8 (61.5%)	0.7475
	T2	≥30% reduction at 48 h	28 (62.2%)	21 (65.6%)	7 (53.8%)	0.4651
Lactate Levels	T1	≥20% reduction at 24 h	20 (44.4%)	16 (50.0%)	4 (30.8%)	0.2446
	T2	≥40% reduction at 48 h	18 (40.0%)	15 (46.9%)	3 (23.1%)	0.1442

Abbreviations: T1, 24 h after therapy initiation; T2, 48 h after therapy initiation. The *p*-value represents the overall significance (Chi-square test).

**Table 4:** 30-Day Mortality.

Source of Infection	Number of Cases (N)	30-Day Mortality (Yes) N (%)	Survival (No) N (%)	*p*-Value
Total Population	45	12 (26.7%)	33 (73.3%)	N/A
Abdominal Source	32	11 (34.4%)	21 (65.6%)	0.0697
Non-Abdominal Source	13	1 (7.7%)	12 (92.3%)	

The *p*-value represents the overall significance (Chi-square test).

**Table 5:** ICU Mortality.

Source of Infection	Number of Cases (N)	Mortality (Yes) N (%)	Survival (No) N (%)	*p*-Value
Total Population	45	14 (31.1%)	31 (68.9%)	N/A
Abdominal Source	32	13 (40.6%)	19 (59.4%)	0.0325
Non-Abdominal Source	13	1 (7.7%)	12 (92.3%)	

The *p*-value represents the overall significance (Chi-square test).

## Data Availability

The data that support the findings of this study are available from the corresponding author, upon reasonable request. Due to the sensitive nature of the data, which includes personal health information and clinical diagnoses, the data are not publicly available to protect the privacy and confidentiality of the research participants.
